# Pseudomonas expression of an oxygen sensing prolyl hydroxylase homologue regulates neutrophil host responses *in vitro* and *in vivo*

**DOI:** 10.12688/wellcomeopenres.12871.1

**Published:** 2017-10-26

**Authors:** Rebecca S. Dickinson, Fiona Murphy, Catherine Doherty, Sam Williams, Ananda Mirchandani, Joseph Willson, John S. Scotti, Gail Preston, Christopher J. Schofield, Moira K.B. Whyte, Sarah R. Walmsley

**Affiliations:** 1 MRC/University of Edinburgh Centre for Inflammation Research, The Queen’s Medical Research Institute, University of Edinburgh, Edinburgh, EH16 4TJ, UK; 2 Chemistry Research Laboratory, Department of Chemistry, University of Oxford, Oxford, OX1 3TA, UK; 3 Department of Plant Sciences, University of Oxford, Oxford, OX1 3RB, UK

**Keywords:** Pseudomonas, neutrophil, hypoxia, Pseudomonas prolyl hydroxylase (PPHD), pyocyanin, apoptosis.

## Abstract

**Background:** Pseudomonas species are adapted to evade innate immune responses and can persist at sites of relative tissue hypoxia, including the mucus-plugged airways of patients with cystic fibrosis and bronchiectasis.  The ability of these bacteria to directly sense and respond to changes in local oxygen availability is in part consequent upon expression of the 2-oxoglutarate oxygenase, *Pseudomonas* prolyl hydroxylase (PPHD), which acts on elongation factor Tu (EF-Tu), and is homologous with the human hypoxia inducible factor (HIF) prolyl hydroxylases. We report that PPHD expression regulates the neutrophil response to acute pseudomonal infection.

**Methods:** *In vitro* co-culture experiments were performed with human neutrophils and PPHD-deficient and wild-type bacteria and supernatants, with viable neutrophil counts determined by flow cytometry. *In vivo* consequences of infection with PPHD deficient *P. aeruginosa* were determined in an acute pneumonia mouse model following intra-tracheal challenge.

**Results:** Supernatants of PPHD-deficient bacterial cultures contained higher concentrations of the phenazine exotoxin pyocyanin and induced greater acceleration of neutrophil apoptosis than wild-type PAO1 supernatants *in vitro.  In vivo* infection with PPHD mutants compared to wild-type PAO1 controls resulted in increased levels of neutrophil apoptosis and impaired control of infection, with higher numbers of *P. aeruginosa* recovered from the lungs of mice infected with the PPHD-deficient strain.  This resulted in an overall increase in mortality in mice infected with the PPHD-deficient strain.

**Conclusions:** Our data show that *Pseudomonas* expression of its prolyl hydroxylase influences the outcome of host-pathogen interactions *in vitro* and *in vivo*, demonstrating the importance of considering how both host and pathogen adaptations to hypoxia together define outcomes of infection. Given that inhibitors for the HIF prolyl hydroxylases are in late stage trials for the treatment of anaemia and that the active sites of PPHD and human HIF prolyl hydroxylases are closely related, the results are of current clinical interest.

## Introduction

*Pseudomonas aeruginosa* is an opportunistic pathogen, which colonizes the airways of patients with chronic inflammatory lung diseases including cystic fibrosis (CF) and bronchiectasis ^1, 2^, and is an important pathogen in the setting of acute ventilation-associated pneumonia ^3^. In the 2004 US CF patient registry, 57% of patients were found to be colonized with *P. aeruginosa* ^4^, whilst children with CF who have sputum positive for *P. aeruginosa* experience more frequent hospitalisation and higher mortality ^5^. These patients have chronic sputum production, with areas of mucus ‘plugging’, resulting in local hypoxia, a condition in which bacteria thrive ^6^. Despite high levels of neutrophilic inflammation, *P. aeruginosa* continues to survive in these patients, evidence that the bacteria employ a number of effective immune-evasion strategies.

A key mechanism by which *P. aeruginosa* impairs host neutrophil function is by generation of phenazine metabolites, particularly pyocyanin, which contributes to the characteristic blue-green colour of infected sputum ^7^. Pyocyanin has previously been shown to accelerate neutrophil apoptosis through activation of the lysosomal death pathway, a process dependent upon the generation of reactive oxygen intermediaries within the neutrophil ^8– 10^ and thus on the availability of molecular oxygen ^11^. More recently, the possibility *Pseudomonas* itself can directly sense and respond to changes in local oxygen availability was raised by the observation that *Pseudomonas spp* contain a 2-oxoglutarate (2OG)-dependent *Pseudomonas* prolyl hydroxylase (PPHD), which acts on the abundant translation elongation factor Tu (EF-Tu) and is homologous to the oxygen sensing hypoxia inducible transcription factor (HIF) prolyl hydroxylase (PHD) enzymes described in eukaryotes ^12^.

Importantly, an insertional mutant strain of *P. aeruginosa* lacking PPHD manifests increased production of pyocyanin under normoxic (room oxygen) standardized broth culture conditions. Moreover, growth of *P. aeruginosa* under conditions of hypoxia has been observed to reduce the pathogenicity of *P. aeruginosa* through repression of production of the siderophores pyoverdine and pyochelin and the secreted virulence factor Exotoxin A ^13, 14^. Thus, the outcomes of host-pathogen interactions may be in part defined by adaptation of both the host and the pathogen to local oxygen availability. In this context, we hypothesised that (1) a PPHD-deficient *P. aeruginosa sp* might demonstrate a survival advantage *in vivo* as a consequence of increased pyocyanin production, leading to accelerated neutrophil apoptosis and impaired neutrophil mediated bacterial killing, and (2) that these effects would be influenced by oxygen availability.

## Methods

### Ethical approval

All participants gave written informed consent in accordance with the Declaration of Helsinki principles, with AMREC approval for the study of healthy human volunteers through the MRC/University of Edinburgh Centre for Inflammation Research blood resource (15-HV-013). Human peripheral blood neutrophils were isolated from whole blood using dextran sedimentation and discontinuous Percoll gradients ^15^.

### Bacterial growth curves

A Columbia blood agar culture plate (VWR International, UK) was inoculated with a single bead from a thawed master stock vial of either wild-type (PA01) or *PA0310* insertional knockout mutant strain (PPHD knockout) pseudomonas and then incubated overnight at 37°C. The following day, ten colonies were taken from the plate using a sterile inoculating loop and used to inoculate 15 ml of sterile Luria-Bertani (LB) broth (Sigma, UK) in a 50ml Falcon tube. The tube was then incubated at 37°C on a shaking platform with the lid loosened. Optical density at 595 nm was measured regularly until plateau.

### Intratracheal pneumonia model

All animal experiments were conducted under an Home Office approved project license in accordance with the Home Office Animals (Scientific Procedures) Act 1986 and University of Edinburgh guidelines in line with the NC3Rs. Six to eight week male C57Bl6J mice were group-housed under standard 12hr light/dark cycles with access to food and water *ad librium*. All efforts were made to ameliorate any suffering of the animals. Mice were closely monitored over the course of the experiments and humanely culled once threshold of severity was reached.

Mice were anaesthetised with ketamine (76mg/kg, Willows Francis Veterinary, UK) and medetomidine (1mg/kg, Orion Pharma, UK) intraperitoneally. Once adequately anaesthetised, the animals were suspended from a frame by the upper incisors and a blunt needle was passed into the trachea via the orotracheal route. Each mouse then had 1×10 ^7^ cfu of either PA01 (wild-type) or PPHD knockout out (mutant) pseudomonas instilled in 50μl PBS via the endotracheal. Twenty minutes after anaesthesia, the mice were given atipamezole (2mg/kg, Orion Pharma, UK), an anaesthetic reversal agent, and recovered for six hours. At indicated time points (6, 12, 24, 36 and 48h after instillation) mice were assessed and tissues harvested. For the Kaplan-Meier plots, mice were culled once the threshold of sickness was reached.

### Assessment of lung injury

Bronchoalveolar lavage (BAL) was obtained by cannulation of the trachea. Total cell counts were calculated using haemocytometer counts and differential cell counts assessed on cytocentrifugation slides. IgM levels were quantified using commercially available kits (Mouse IgM ELISA quantitation set, Bethyl Laboratories Inc, Montgomery, USA; EnzChek Elastase Assay Kit, Molecular Probes Europe BV, Leiden, The Netherlands).

For histological analysis, lungs were fixed with 10% buffered formalin and embedded into paraffin blocks. Tissues slices were fixed and stained with haematoxylin and eosin.

### Flow cytometry for BAL neutrophil apoptosis

BAL cells were counted and 1×10 ^6^ cells were centrifuged at 300g for 5 minutes at 4°C. Cell pellets were resuspended in 50uL of FC block (1:100 anti-CD16/32 Ab, **RRID:AB_312801**; Biolegend) and 1:10 mouse serum in FACS buffer (PBS with 0.5% BSA and 0.02mM EDTA) and incubated on ice for 15 minutes. Subsequently, cells were stained with 50ul anti-Ly6G Ab ( **RRID:AB_1236494**; BioLegend) at 1:200 final concentration and incubated on ice for 30 minutes in the dark. Following a wash with FACS buffer and centrifugation at 300g for 5 minutes at 4°C, cells pellets were resuspended in Annexin-binding buffer and Annexin-V PE stain (Becton Dickinson) for 15 minutes at room temperature in the dark. Prior to flow cytometry acquisition, cells were stained with Topro3 APC (Molecular Probes). Neutrophils were gated based on Ly6G expression and Annexin-V and Topro3 expression was quantified.

Cells were acquired using a BD Calibur machine and analysed using FlowJo version 10 software (Tree Star).

### Quantification of viable bacterial counts

10-fold serial dilutions were performed on whole blood aliquots and lungs homogenized in sterile tubes following collection of BAL fluid. Three 10μl drops from each of 6 dilutions were then plated onto blood agar plates and cultured overnight in 37°C to calculate viable bacterial counts, which were normalized to count per ml of blood or per pair of lungs.

### Production of bacterial supernatants

Ten colonies were taken from the blood agar culture plate and used to inoculate plates containing 20ml of pseudomonas isolation agar (Difco). These plates were incubated overnight at 37°C and then placed in direct sunlight for 48h to allow pigment to develop. Each plate was then flooded with 6ml RPMI media (Sigma, UK) and left at room temperature for 2 hours. The RPMI was removed, spun at 4000g for 15 min, twice, and filter sterilised through a 0.22μm filter to remove any bacteria. To ensure sterility, 100μl of each supernatant was used to inoculate a blood agar plate and cultured for 48h at 37°C. Supernatants were stored at -80°C.

### Quantification of pyocyanin concentration

PPHD mutant and wildtype colonies were inoculated into 10 ml of LB broth and incubated overnight at 37°C in a shaking incubator. 1ml of the overnight cultures were then inoculated into 9ml of LB broth and incubated for 2 hours at 37°C in a shaking incubator. 100 μl of each strain was then pipetted onto Pseudomonas Isolation agar (Difco) plates and incubated overnight under conditions of normoxia (21% O _2_) and hypoxia (3% O _2_) and supernatants produced as detailed above. 4.5ml of chloroform was added to 7.5ml of sterile bacterial supernatant and vortexed. Samples were centrifuged at 2000g for 10 minutes. 3ml of the chloroform layer was transferred to a clean tube and 1.5ml 0.2M hydrochloric acid was added. Tubes were vortexed and spun at 2000g for 2 minutes. 1ml of the top layer was removed, absorbance at 520nm measured and pyocyanin concentrations determined ^16^.

### Isolation and culture of human neutrophils

Human peripheral blood neutrophils were isolated from whole blood using dextran sedimentation and discontinuous Percoll gradients. Neutrophils were resuspended in RPMI with 20% fetal calf serum (Lifetech, Paisley, UK) at 10x10 ^6^/ml. 75μl of this suspension was cultured with 75μl of either wild-type (PA01) or mutant (PPHD knockout) pseudomonas supernatant for five hours in either normoxia (room air) or hypoxia (1% oxygen, *in vivo* 400 hypoxia workstation, Ruskinn). After 5 hours, cells were removed from the culture plate and pelleted at 400g for 5 minutes. The pellets were resuspended in 95μl annexin binding buffer and 5μl annexin V/PE (Becton-Dickinson) and incubated on ice for 20 minutes. 100μl of Topro3/APC (Molecular Probes) and 5×10 ^4^ Countbright™ absolute counting beads (ThermoFisher, UK) were added to each sample, and samples run using a BD FACSCalibur (BD Biosciences, UK).

### Statistical analysis

Data were analysed using Prism 7.0 software (GraphPad Software Inc., San Diego, CA). Unpaired t-tests were used for comparisons between wild-type and knockout sample means. Two-way ANOVA with Bonferroni’s post-test comparisons was performed if multiple time points were used. For comparison of viable bacterial counts, Mann-Whitney test was performed. Survival was analysed using log-rank test. Statistical significance was accepted when p<0.05.

## Results

### Neutrophil co-culture with PPHD mutant bacterial supernatants induced cell loss, which was reversed with hypoxic culture

**Figure 1.**
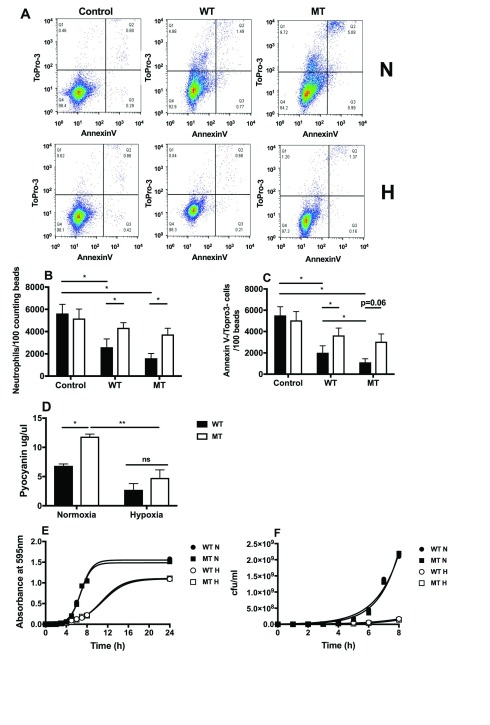
Supernatants from *Pseudomonas* prolyl hydroxylase (PPHD) knockout *P. aeruginosa* induce neutrophil death via increased production of pyocyanin. Human neutrophils were cultured with PA01 wildtype (WT) or PPHD knockout (MT) bacterial supernatant for 5 hours in normoxia (N; filled bars) or hypoxia (H; open bars). Flow cytometry ( **A**) was performed to calculate total ( **B**) and viable neutrophil numbers ( **C**). n= 5 *p<0.05. ( **D**) Pyocyanin concentrations in supernatants from wildtype (WT) and PPHD knockout *P. aeruginosa* (MT) in normoxia and hypoxia were measured. n=3, *p<0.05, **p<0.01. Wildtype (WT) and PPHD knockout *P. aeruginosa* (MT) were grown in normoxia (N, 21% oxygen) and hypoxia (H, 1% oxygen). Absorbance at 595nm ( **E**) and viable bacterial count ( **F**) were recorded to plot growth curves.

To directly address whether expression of the hypoxia sensing prolyl hydroxylase PPHD by *P. aeruginosa* would affect rates of neutrophil apoptosis, freshly isolated human peripheral blood neutrophils were cultured for 5h with sterile supernatants harvested from wild type PA01 and PPHD-deficient bacterial cultures *in vitro,* a time-point at which pyocyanin markedly accelerates neutrophil apoptosis ^8^. Total neutrophil numbers and neutrophil viability were assessed by flow cytometry ( Figure 1A). In normoxia, both PA01 and PPHD-deficient supernatants caused significant loss of neutrophil numbers ( Figure 1B) and a reduction in Annexin V- /Topro 3- (viable) neutrophils recovered. Greater reductions in viable cell numbers (Annexin V-/Topro 3-) were observed when neutrophils were cultured in the presence of PPHD-deficient supernatants ( Figure 1C). Hypoxic cell culture reversed the increases in both cell loss and apoptosis observed with PAO1 and PPHD-deficient supernatants ( Figure 1A–C), in keeping with the dependence of pyocyanin-induced apoptosis on the availability of oxygen ^11^.

To address whether the observed differences in neutrophil loss reflected altered pyocyanin production, we measured pyocyanin production over a 48 hour inoculation of blood agar following recovery into RPMI media. Under conditions of normoxia (21% O _2_), the PPHD-deficient strain produced significantly higher levels of pyocyanin than the wild-type strain ( Figure 1D), in keeping with the greater loss of viable neutrophil numbers ( Figure 1C). Elevated pyocyanin production by the PPHD-deficient strain was abrogated entirely by use of a hypoxic (1% O _2_) cell culture ( Figure 1D). This was not a consequence of differential bacterial growth rates, with equivalent 595 nm absorbance and bacterial counts being observed for PA01 and PPHD mutants at each oxygen tension studied ( Figure 1E, F). Hypoxia (1% O _2_), whilst impairing bacterial growth, had no differential growth effects on PAO1 compared with the PPHD-deficient strain.

### PPHD-deficient *P. aeruginosa* infection results in increased mortality and lung injury during acute pneumonia and greater impairment of neutrophil-mediated host defense compared with wild-type PAO1 infection

**Figure 2.**
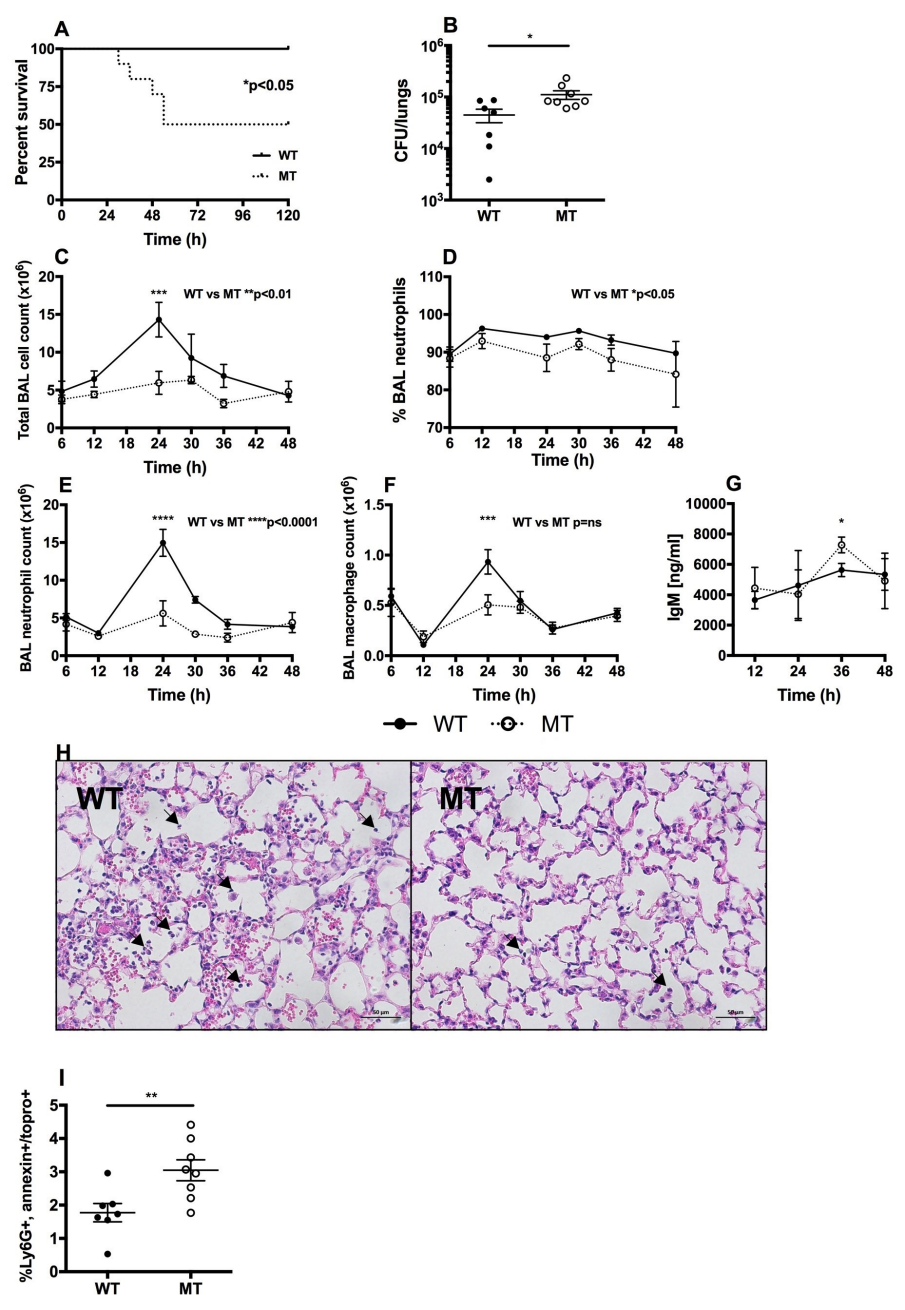
Infection with *Pseudomonas* prolyl hydroxylase (PPHD) knockout *P. aeruginosa* carries higher mortality. C57/BL6 mice were instilled intratracheally with 1×10 ^7^ cfu of PA01 wildtype (WT) or PPHD knockout (MT) *P. aeruginosa.* ( **A**) Survival was recorded for 5 days post infection, n=10 mice per group. ( **B**) At 12 hours post infection lungs were harvested and viable bacteria count calculated, n=8 mice per group *p<0.05. Bronchoalveolar lavage (BAL) total cell count ( **C**), % neutrophils ( **D**), neutrophil count ( **E**), macrophage count ( **F**) and BAL supernatant immunoglobulin M (IgM) ( **G**) were measured at timepoints from 12–48 hours post infection, n= 4–7 mice per group, *p<0.05, ***p<0.001, ****p<0.0001. ( **H**) H+E staining of lung tissue taken at 36h after infection (arrows point to neutrophils, x20 magnification). Images representative for n=2 mice per group. ( **I**) BAL was harvested and the cell pellets analysed by flow cytometry for apoptosis at 12 hours post infection, n=8 mice per group **p<0.01.

To define whether *PPHD* deficiency results in an altered course of acute *P. aeruginosa* infection *in vivo*, mice were challenged via the trachea with 1×10 ^7^ cfu PAO1 (wildtype) or PPHD-deficient bacteria. 50% of animals receiving PPHD-deficient *P. aeruginosa* reached sickness thresholds requiring the animals to be culled by day 5 ( Figure 2A). In contrast, all PAO1 infected mice were viable up to 5 days following infection challenge ( Figure 2A, *p<0.05). Importantly, this increase in mortality was associated with a 2.5-fold greater bacterial burden in the lungs of PPHD mutant infected (11.1×10 ^4^CFU/lung± 5.99×10 ^4^) compared to wildtype infected mice (4.48×10 ^4^CFU/lung ± 3.48×10 ^4^, Figure 2B, p<0.05).

In light of the equivalent growth of wild-type and PPHD mutant strains we had observed *in vitro*, we questioned whether the increase in bacterial numbers in PPHD mutants was a consequence of an impaired host response. Whilst the initial recruitment of inflammatory cells to the lungs (6h and 12h) was similar between wildtype and *PPHD*-deficient *P. aeruginosa* infected mice ( Figure 2C–F), significantly fewer cells were recovered from the airways of PPHD-deficient infected mice by 24h after infection ( Figure 2C, **p<0.01), as a consequence of reductions in both the percentage ( Figure 2D, *p<0.05) and total number of airway neutrophils ( Figure 2E and F, **** p<0.0001). In keeping with a more severe infection in PPHD-deficient infected mice, higher levels of IgM, an indirect marker of vascular leak and lung injury, were detected in mice infected with mutant PPHD ( Figure 2G, *p<0.05). Histological analysis of lungs of mice infected with *P. aeruginosa* supported the observed differences in BAL, with fewer neutrophils in the lungs of mice infected with mutant *PPHD* ( Figure 2H).

In light of the increased production of pyocyanin by PPHD mutant *P. aeruginosa* and the observed increase in neutrophil loss with PPHD supernatants, we hypothesised the reduction in neutrophil numbers observed at 24 hours to be a consequence of increased levels of neutrophil apoptosis. Ly6G+ airway recovered neutrophils were therefore dual stained with Annexin V/Topro3 to directly quantify the number of apoptotic cells following Pseudomonas infection. Infection with mutant PPHD *P. aeruginosa* resulted in higher detectable levels of apoptosis than infection with the PAO1 wild type strain ( Figure 2I, p<0.01).

## Discussion

A significant focus of research from our group and others has centered round defining the mechanisms by which hypoxia directly regulates immune cell function ^17– 22^. Innate responses to bacterial challenges are critically regulated by oxygen availability, with neutrophils in particular being adapted to survive in hypoxic tissues where they phagocytose and kill bacteria ^11, 21, 23^. Until recently, the possibility that oxygen may also regulate the behavior of bacterial pathogens has not been considered. This is of particular relevance to *Pseudomonas spp* that persist in chronically inflamed tissues characterized by limited oxygen availability ^24^ and induce oxidant-dependent cell death via the production of the phenazine, pyocyanin ^10, 25^. The results described here reveal the importance of the *Pseudomonas* prolyl hydroxylase, PPHD, in regulating the effectiveness of neutrophil mediated host defenses *in vivo*, likely, at least in part, as mediated by variations in the levels of the toxic metabolite pyocyanin.

Suppression of PHD activity is described in eukaryotic systems in the context of hypoxia – indeed is central to regulation of the hypoxic response ^26– 30^. Diminished *Pseudomonas aeruginosa* pathogenicity in hypoxia has recently been described as a consequence of reduced expression of the virulence factors pyoverdine and exotoxin A ^14^, with our work extending this to include production of pyocyanin. This is of interest, given that neutrophil respiratory burst activity, a key anti-microbial defence, is associated with promotion of a hypoxic niche ^22^. It is also of relevance to the oxygen requiring process by which the pyocyanin induces ROI-mediated lysosomal dysfunction and neutrophil apoptosis ^10^, as evidenced by the reduction in cell loss we observed when neutrophils were challenged with *P. aeruginosa* conditioned media in the context of hypoxia. Thus in clinical scenarios in which tissue oxygen availability is severely limited, oxygen dependent regulation of the balance between innate immune responses and bacterial virulence and replicative capacity may be critical in defining the outcomes of infection and host morbidity and mortality. This is, however, further complicated by the observations that differential expression of oxygen sensing prolyl hydroxylase enzymes either by immune cells ^20, 31, 32^, or bacterial pathogens ^12^ can also directly regulate cellular function and bacterial virulence when oxygen is not a limiting factor. For example, neutrophil loss of PHD2 under physiological normoxia promotes a phenotype of excessive neutrophilic inflammation ^33^, whilst deletion of PPHD is associated with increased production of pyocyanin ^12^. *In vivo* therefore, the dominant phenotype is likely to be in part determined by the physiological Κ _m_, in which both PPHDs and PHDs function in both innate immune and bacterial cells. Of interest, kinetic analysis of the isolated PPHD enzyme has identified a lower apparent Κ _m_ for O _2_ than PHD2, but a higher Κ _m_ for Fe(II), suggesting that iron regulation may also be of critical importance in defining the activity of PPHD enzyme activity in a physiological setting ^12^. This is of particularly relevance to *Pseudomonas spp* where enhanced iron redox states enable competitive outgrowth from other bacterial species ^34^.

In this work, we provide *in vivo* data, describing the clinical outcomes when mice are challenged with acute *P. aeruginosa* infection in the context of normal lung architecture and therefore relatively preserved local tissue oxygenation. In this setting we observe increased mortality with PPHD-deficient strains as a consequence of insufficient neutrophil host defense and failure to control bacterial replication. Thus, we can now extend the concept that immune cell loss of PHD2 in the context of preserved tissue oxygenation promotes a detrimental immune response to also include detrimental consequences of prokaryotic loss of PPHD expression. This has potentially important ramifications in light of the current development of relatively non-selective PHD inhibitors (which may well inhibit PPHD), as well as the use of iron chelators in the clinical arena, and how they may impact more widely on the host pathogen response with consequence both for the host and the pathogen.

## Data availability

The data referenced by this article are under copyright with the following copyright statement: Copyright: © 2017 Dickinson RS et al.

Raw data counts available via Figshare: https://doi.org/10.6084/m9.figshare.5484178.v1 ^35^
