# Supplementary material for: Pseudomonas expression of an oxygen sensing prolyl hydroxylase homologue regulates neutrophil host responses in vitro and in vivo
Source: Wellcome Open Res. 2017 Oct 26;2:104. [Version 1] doi: 10.12688/wellcomeopenres.12871.1 (PMC5701443; doi:10.12688/wellcomeopenres.12871.1)
Supplement: Peer review history

# Peer review: Referee response for version 1

**DOI:** 10.21956/wellcomeopenres.13951.r28124

**Published:** 2017-12-07

**Authors:** Stilla Frede

**Affiliations:** 1 Department of Anesthesiology and Intensive Care Medicine, University Hospital Bonn, Bonn, Germany

In this manuscript the authors clearly describe the fatal effects of PPHD knockout for the clinical outcome of mice challenged with this bacterial strain. The data are convincing and point to the problems possibly occurring with the use of small molecule inhibitors of PHDs.

 I have a question regarding the signalling pathway(s) underlying the increased Pyocyanin production in PPHD knockout bacteria. If the classical inhibition of PHD activity under hypoxic conditions is involved why is the production of Pyocyanin not increased in the wt strain under these conditions?

I have read this submission. I believe that I have an appropriate level of expertise to confirm that it is of an acceptable scientific standard.

# Peer review: Referee response for version 1

**DOI:** 10.21956/wellcomeopenres.13951.r27891

**Published:** 2017-11-22

**Authors:** Benoît S. Marteyn

**Affiliations:** 1 Pasteur Institute, Molecular Microbial Pathogenesis, Paris, France

In this manuscript, Dickinson and colleagues reveal the impact of P. aeruginosa PPHD on the bacteria virulence using in vitro and in vivo models. In particular, the PPHD mutation-dependent  increased concentration of pyocyanin in bacterial supernatants was characterized and associated with increased neutrophil apoptosis. The manuscript is clear, well written and the presented data support the conclusions.

 In order to better appreciate the contribution of pyocyanin on P. aeruginosa mediated neutrophil apoptosis, the following points should be addressed:

1. The authors studied the impact of pyocyanin on neutrophil survival using bacterial supernatants form WT and PPHD-deficient mutant. In order to evaluate the contribution of pyocyanin on P. aeruginosa-dependent neutrophil apoptosis induction, the authors should additionally infect neutrophils with WT and PPHD-deficient mutant strains and assess neutrophil viability (w/wo oxygen). The comparison with neutrophil apoptosis levels induced with bacterial supernatant should be then discussed.
2. It would be informative to assess the ability of neutrophils to kill WT and MT strains (w/wo oxygen) and the results should be discussed to better interpret in vivo results.
3. In the discussion, the authors say that "the possibility that oxygen may also regulate the behavior of bacterial pathogens has not been considered”. This statement is not true, additional references should be included regarding the O2-modulation of bacteria virulence, adhesion, secretion, etc.

I have read this submission. I believe that I have an appropriate level of expertise to confirm that it is of an acceptable scientific standard, however I have significant reservations, as outlined above.

# Response

**Published:** 2017-11-22

**Authors:** Sarah Walmsley

**Affiliations:** University of Edinburgh, UK

We thank Dr Marteyn for the review of our manuscript.

1. In vitro co-culture of neutrophils directly with pseudomonas species rapidly results in neutrophil loss. This is why we firstly explored the consequence of neutrophil culture with bacterial supernatants before undertaking in vivo experiments in which we were able to directly address the consequence of infection with the different strains of P. aeruginosa on neutrophil survival in a biological setting. 
2. Due to the ability of pseudomonas to induce neutrophil apoptosis, this is a difficult question to directly address.  We would however argue that it is the ability of Pseudomonas to evade the host response that is critical in defining the outcome of the infection challenge, a concept supported by the published literature (Usher et al. JI 2002; Allen et al. JI 2005; Prince et al. JI 2008) and our in vivo observations.
3. We are sorry for any confusion caused, in the discussion we do actually state that “until recently, the possibility that oxygen may also regulate the bacterial pathogens has not been considered” and provide a number of references specific to Pseudomonas throughout the text that reference that capacity of hypoxia to alter pathogenicity (Scotti et al. PNAS 2014; Schaible et al. PLOS One 2013; Schaible et al. J Infect Dis 2017).

# Peer review: Referee response for version 1

**DOI:** 10.21956/wellcomeopenres.13951.r28123

**Published:** 2017-11-22

**Authors:** Andrew S. Cowburn

**Affiliations:** 1 Department of Physiology, Development and Neuroscience, University of Cambridge, Cambridge, UK; 2 Department of Medicine, University of Cambridge, Cambridge, UK

The authors very elegantly describe a key role for Pseudomonas prolyl hydroxylase (PPH) in the sensing of oxygen tension and the release of the phenazine exotoxin pyocyanin, this enhances the rate of neutrophil apoptosis and compromises the host innate immune response. The authors competently used two model systems, human neutrophil incubated in the presence of supernatants from wt Pseudomonas or mutant lacking PPH cultured in either normoxia or hypoxia, and a murine pneumonia model. Both systems clearly show that infection with Pseudomonas deficient in PPH results with increased neutrophil cell death and compromised host response to the infection resulting in increased mortality in the murine model.

 I have one minor question regarding the murine model used. Fig 2E shows a significant drop in BAL neutrophil number at 24hrs, however in Fig 2I the authors analysed BAL from the 12hr time point showing a small but significant shift in neutrophil cell death. The description in the results refers to BAL 24hr data set. If the data is available from 24hr BAL I believe this would enhance Fig 2 and benefit the readers understanding.

 This manuscript further highlights the importance of understanding how the new generation of small molecular inhibitors that interact with the oxygen sensing pathway need to be comprehensively investigated not only at the cellular/tissues level but also at the point of host pathogen interaction.

I have read this submission. I believe that I have an appropriate level of expertise to confirm that it is of an acceptable scientific standard.

# Response

**Published:** 2017-11-22

**Authors:** Sarah Walmsley

**Affiliations:** University of Edinburgh, UK

We thank Dr Cowburn for highlighting the importance of the observations detailed in the manuscript.  With respect to the time points studied, given we were already detecting a marked difference in BAL neutrophil counts at 24 hours, and that apoptotic cells are rapidly cleared in the in vivo setting, we chose to study a time point preceding one associated with significant cell loss (12 hours), to enable us to measure changes in surface apoptosis markers.

# Peer review: Referee response for version 1

**DOI:** 10.21956/wellcomeopenres.13951.r27357

**Published:** 2017-11-15

**Authors:** Susanne Schlisio

**Affiliations:** 1 Department of Cell and Molecular Biology, Ludwig Institute for Cancer Research, Karolinska Institute, Stockholm, Sweden

The authors provide convincing *in vivo* evidence that prolyl hydroxylase-decient *P. aeruginosa* infection results in increased levels of neutrophil apoptosis, impaired control of infection, and consequently increased mortality in mice. The authors conclude that the expression of the oxygen sensing prolyl hydroxylase homologue (PPHD) in Pseudomonas regulates neutrophil host responses *in vivo*. The importance of this finding is in light of current development of relatively non-selective PHD inhibitors that most likely inhibit both, the host and Pseudomonas prolyl hydroxylase and thus impact both, host and pathogen.

 The *in vivo* experiments and analyses presented in this work appear robust and of high quality.

 I only have a minor comment regarding *in vitro* studies in Fig 1B:

 It seems that under normoxia, there was no significant change in neutrophil numbers cultured with WT or PPHD mutant supernatant (Fig 1B normoxia -black bar- WT versus MT). Since pyocyanin was significantly increased in the MT under normoxia (Fig 1D), why was that not reflected in significant decrease of neutrophil counts under normoxic conditions in MT versus WT (FIG 1B)? Please provide comments. What is the p value: WT versus MT under normoxia in Fig 1B? Is the mild reduction of neutrophil counts significant under normoxia (WT vs MT)?

I have read this submission. I believe that I have an appropriate level of expertise to confirm that it is of an acceptable scientific standard.

# Response

**Published:** 2017-11-16

**Authors:** Sarah Walmsley

**Affiliations:** University of Edinburgh, UK

We thank Dr Schlisio for her careful review of our manuscript and comments. Although the mean number of neutrophils following co-culture with mutant supernatants is lower than wildtype (1610±426 MT vs 2591±749) this does not reach statistical significance (P=0.89 by two way ANOVA).  We attribute this to significant cell loss with both wildtype and mutant supernatants under normoxic culture conditions given both strains of P. aeruginosa produce toxic levels of pyocyanin in normoxia.
